# Recent introductions reveal differential susceptibility to parasitism across an evolutionary mosaic

**DOI:** 10.1111/eva.12865

**Published:** 2019-09-25

**Authors:** Carolyn K. Tepolt, John A. Darling, April M. H. Blakeslee, Amy E. Fowler, Mark E. Torchin, A. Whitman Miller, Gregory M. Ruiz

**Affiliations:** ^1^ Department of Biology Woods Hole Oceanographic Institution Woods Hole MA USA; ^2^ Smithsonian Environmental Research Center Edgewater MD USA; ^3^ National Exposure Research Laboratory US Environmental Protection Agency Research Triangle Park NC USA; ^4^ Department of Biology East Carolina University Greenville NC USA; ^5^ Department of Environmental Science and Policy George Mason University Fairfax VA USA; ^6^ Smithsonian Tropical Research Institute Balboa Ancon Republic of Panama

**Keywords:** adaptation in invasion, biological introductions, host–parasite evolution, *Loxothylacus panopaei*, mud crabs, parasite biogeography, *Rhithropanopeus harrisii*, rhizocephalans

## Abstract

Parasitism can represent a potent agent of selection, and introduced parasites have the potential to substantially alter their new hosts' ecology and evolution. While significant impacts have been reported for parasites that switch to new host species, the effects of macroparasite introduction into naïve populations of host species with which they have evolved remain poorly understood. Here, we investigate how the estuarine white‐fingered mud crab (*Rhithropanopeus harrisii*) has adapted to parasitism by an introduced rhizocephalan parasite (*Loxothylacus panopaei*) that castrates its host. While the host crab is native to much of the East and Gulf Coasts of North America, its parasite is native only to the southern end of this range. Fifty years ago, the parasite invaded the mid‐Atlantic, gradually expanding through previously naïve host populations. Thus, different populations of the same host species have experienced different degrees of historical interaction (and thus potential evolutionary response time) with the parasite: long term, short term, and naïve. In nine estuaries across this range, we examined whether and how parasite prevalence and host susceptibility to parasitism differs depending on the length of the host's history with the parasite. In field surveys, we found that the parasite was significantly more prevalent in its introduced range (i.e., short‐term interaction) than in its native range (long‐term interaction), a result that was also supported by a meta‐analysis of prevalence data covering the 50 years since its introduction. In controlled laboratory experiments, host susceptibility to parasitism was significantly higher in naïve hosts than in hosts from the parasite's native range, suggesting that host resistance to parasitism is under selection. These results suggest that differences in host–parasite historical interaction can alter the consequences of parasite introductions in host populations. As anthropogenically driven range shifts continue, disruptions of host–parasite evolutionary relationships may become an increasingly important driver of ecological and evolutionary change.

## 1 INTRODUCTION

Biological invasions can be profoundly destabilizing to native ecosystems, in part because they disrupt established biotic relationships and alter species interactions. One understudied aspect of this invasion‐mediated change in species interactions is the impact of introduced parasites on naïve native hosts. Parasites are an integral part of ecological communities and play a key role in community structure and food web stability (Lafferty, Dobson, & Kuris, 2006; Wood et al., 2007). Under strong and prolonged parasite pressures, hosts may evolve physiological traits that lower their infection susceptibility or behavioral traits that allow them to escape from parasitism (Bérénos, Schmid‐Hempel, & Wegner, 2009; Duncan & Little, 2007; Hart, 1990; Tolley, Winstead, Haynes, & Volety, 2006). In turn, parasites may evolve to better infect and exploit their hosts (Little, Watt, & Ebert, 2006). Thus, the introduction of novel parasites can have important impacts on native hosts and, in turn, on their communities, by altering these evolutionary associations (Britton, 2013; Loo, 2009).

As rates of species introduction accelerate due to increased globalization, so does the potential for the concomitant introduction of novel parasites (Ruiz, Fofonoff, Carlton, Wonham, & Hines, 2000; Telfer & Bown, 2012). While introduced species leave behind many of their parasites in the invasion process, they seldom lose all of them (Blakeslee, Fowler, & Keogh, 2013; Torchin, Lafferty, Dobson, McKenzie, & Kuris, 2003; Torchin & Mitchell, 2004). Introduced parasites, in turn, can spill over to naïve hosts in the local community (Lymbery, Morine, Kanani, Beatty, & Morgan, 2014; Tompkins, Dunn, Smith, & Telfer, 2011). Much of the literature on introduced parasites focuses on native host and exotic parasite as strangers to one another—that is, host‐switching by the introduced parasite to exploit a novel host species (Goedknegt et al., 2016; Tompkins et al., 2011). In contrast, there has been very little exploration of parasite spillover *without* host‐switching, where the parasite is transported from its native region to an area harboring naïve populations of the same host species (Woolhouse, Webster, Domingo, Charlesworth, & Levin, 2002). In this scenario, the parasite will likely have a distinct advantage because it has adapted to infect the host species, while the naïve host population has not had the opportunity to evolve resistance to parasitism.

Laboratory studies have demonstrated that intraspecific differences in the interaction history of both host and parasite can significantly influence susceptibility to infection (Gibson, Jokela, & Lively, 2016; Webster & Woolhouse, 1998) and that host populations can evolve rapidly under strong parasite‐induced selective pressures (Webster & Woolhouse, 1998; Zuk, Rotenberry, & Tinghitella, 2006). A larger body of work on host–parasite evolutionary dynamics has focused on microbial parasites and pathogens, notably the explosion of smallpox and other diseases in unexposed human populations which contributed to sweeping changes in human culture and colonization (Crosby, 2004; Fenner, 1993). However, intraspecific differences in susceptibility may exist in any system where the host and parasite distributions do not fully overlap, for example, when a host is more widespread geographically than its parasites. Such a scenario can then result in a mosaic of host–parasite relationships across a host's range. In many systems, macroparasite communities are so understudied that the potential influence of invasions resulting from such mismatches in host and parasite ranges is typically overlooked (Vignon & Sasal, 2010).

In this study, we tested the impact of host–parasite evolutionary history on the prevalence of the parasite in the wild and on the host's susceptibility to parasitism under controlled laboratory conditions. We used a system with two widespread host crab species and a castrating barnacle parasite, in which the parasite (*Loxothylacus panopaei*) has a more restricted native range than its hosts (*Rhithropanopeus harrisii* and *Eurypanopeus depressus*). Given the strong selective pressure exerted by the parasite on the host (permanent castration), this system is likely characterized by longstanding coevolution between host and parasite (Ashby & Gupta, 2014). However, we note that this study is focused on potential host evolution in response to the parasite and does not consider evolution of the parasite itself. The introduction and subsequent spread of this parasite in naïve host populations outside of the parasite's native range offer a natural test of the potential effects of interaction history on the host's evolutionary ecology. As the host on which we primarily focus (*R. harrisii*) is itself a widespread introduced species, we also discuss the potential implications should the parasite be introduced to newly established host populations around the world.

We hypothesized that the crab host is evolving in response to the parasite in its native range, given the strong selective pressure of permanent castration as a consequence of parasitism, and that crabs without a long‐term history with the parasite would be more susceptible to parasitism. We tested two specific predictions based on this hypothesis, using data from a field survey, a literature survey, and a controlled laboratory experiment. First, we tested whether the parasite was more prevalent in its invasive range than in its native range. We conducted a widespread field survey spanning more than 4,000 km of shoreline along eastern North America, comparing host demography and parasite prevalence among estuaries where the parasite is native, introduced, and absent. Concurrently, we conducted a meta‐analysis of reported parasite prevalence in host crabs for the same geographic region, incorporating our newly collected data. The empirical survey data were conducted using a standardized approach and focused on *R. harrisii*, which has been substantially understudied compared with the other host species. The meta‐analysis was used to compare parasite prevalence between the native and introduced ranges in both hosts, drawing on over 200 records collected since 1964 to ensure that our conclusions were robust. Next, we conducted laboratory experiments under controlled conditions to test whether crabs from the parasite's native range were less susceptible to parasitism than entirely naïve crabs and crabs from the parasite's invasive range. Our results highlight the importance of host–parasite interaction history in shaping the ecological and evolutionary outcomes of parasite introductions.

## 2 MATERIALS AND METHODS

### 2.1 Host–parasite study system

Rhizocephalans are parasitic barnacles that infect decapod crustacean hosts; they have direct transmission with a brief free‐living larval stage (Høeg, 1995). Rhizocephalans infecting brachyurans alter host behavior, feminize male hosts, and castrate both male and female hosts (Reinhard, 1956; Shields, Williams, & Boyko, 2015). Infection with the rhizocephalan *Loxothylacus panopaei* has been shown to alter host feeding and activity, increase susceptibility to predation, and change the structure of the larger ecological community (Belgrad & Griffen, 2015; Eash‐Loucks, Kimball, & Petrinec, 2014; Gehman & Byers, 2017; O’Shaughnessy, Harding, & Burge, 2014; Toscano, Newsome, & Griffen, 2014). While *L. panopaei* was traditionally identified as a single parasite species that infects several panopeid crab species, recent molecular work has identified deep genetic divides within this taxonomic designation. *Loxothylacus panopaei* likely represents a cryptic species complex comprised of at least two to three parasite species with distinct host spectra (Kruse & Hare, 2007; Kruse, Hare, & Hines, 2011). Since the “*L. panopaei*” designation has not yet been formally reclassified, here unless otherwise specified, we use “*L. panopaei*” to refer to one specific clade (the ER clade) that infects *Eurypanopeus depressus* and *Rhithropanopeus harrisii* (Kruse et al., 2011).

**Figure 1:**
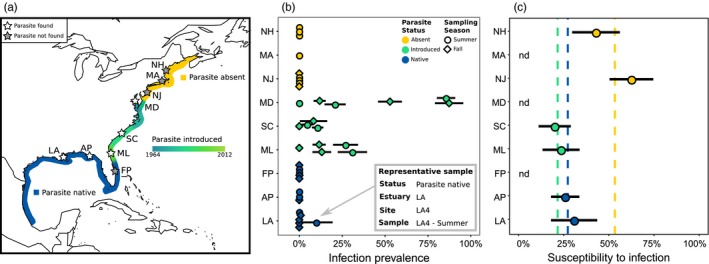
(a) Map of invasion history and sampled estuaries (stars); additional details are given in Table 1. In addition to the contiguous introduced range in the central and southern Atlantic coast of North America, there is a highly restricted and recently introduced population in Long Island Sound (first reported in 2012). (b) Prevalence of *Loxothylacus panopaei* infection in *Rhithropanopeus harrisii* in 2015 field surveys. Each point represents one site × time sample; details given for one representative sample in inset box. Samples with <10 adult crabs are not shown. Points represent prevalence of external infection in individuals >3.9 mm CW; bars indicate standard error. Points are jittered vertically for clarity. (c) *R. harrisii* susceptibility to *L. panopaei* parasitism in controlled laboratory exposures. Points indicate proportion of exposed crabs infected, with bars showing standard error. Dashed lines indicate overall susceptibility for each regional parasite status (native, introduced, and absent). Raw data are presented in Table 3

Both hosts, *R. harrisii* and *E. depressus*, have wide native ranges spanning much of the Atlantic and Gulf coasts of North America (Williams, 1984). In contrast, the parasite *L. panopaei* was historically restricted to the Gulf Coast and south of Cape Canaveral, Florida (Hines, Alvarez, & Reed, 1997; Kruse et al., 2011; Figure 1a). The parasite invaded the Chesapeake Bay in the early 1960s, likely introduced via infected host crabs associated with live oyster shipments intentionally transported from the Gulf Coast after the collapse of the Chesapeake Bay oyster fishery (Andrews, 1980; Van Engel, Dillon, Zwerner, & Eldridge, 1966). Once in the Chesapeake Bay, the parasite rapidly spread south, finally connecting with its suspected native range boundary at Cape Canaveral (Hines et al., 1997; Kruse et al., 2011). Recently, an isolated population has been discovered in Long Island Sound, where it is believed to have been locally introduced in the course of oyster restoration efforts or via ballast water (Freeman, Blakeslee, & Fowler, 2013; Kroft & Blakeslee, 2016). Currently, with the exception of the geographically restricted Long Island population, *L. panopaei* has not been observed north of the Chesapeake Bay (A. M. H. Blakeslee & C. K. Tepolt, personal communication).

### 2.2 Field surveys

We sampled host crab populations that have experienced different degrees of interaction history with the parasite *L. panopaei*, focusing primarily on the more brackish host, *R. harrisii.* Sampling was conducted in nine estuarine systems spanning the native range of *R. harrisii* along the East and Gulf Coasts of the USA (Figure 1a, Table S1.1). Sampled estuaries were distributed among regions with distinct histories of *L. panopaei* parasitism: three estuaries where the parasite is native (Louisiana, Gulf Coast Florida, and southern Atlantic Florida), three estuaries where the parasite is introduced (northern Atlantic Florida, South Carolina, and Maryland), and three estuaries where the parasite has not yet invaded and crab hosts are thus completely naïve to the parasite (New Jersey, Massachusetts, and New Hampshire). Because estuarine systems are highly dynamic, sampling was carried out at three to four sites per estuary located along a salinity gradient (~5–25 Practical Salinity Units, PSU) to capture the environmental conditions where the brackish *R. harrisii* is most frequently found (Williams, 1984). Salinity and temperature were measured with a handheld YSI Pro30 meter (YSI, Yellow Springs, OH) at the time of sampling.

At each site, two passive “crab collectors” were deployed on the benthos in 0.5‐4 m water depths. Collectors do not trap animals but rather mimic natural habitat, providing a refuge for colonization by the crabs and serving as a standardized sampling method across locations. Collectors were initially deployed between 4 June and 3 July 2015, capturing the host's breeding and recruitment season (Goy, Morgan, & Costlow, 1985). Most collectors were sampled twice, once in the mid‐late summer and once in the early autumn (Table S1.1), following a minimum deployment duration of 4 weeks. Collectors from MA and NH were checked once, in the late summer, to collect data on host populations in the absence of parasitism. During each check, all panopeid crabs ≥2 mm carapace width (CW) were collected and later examined under a dissecting microscope for the following: species identity, CW (in mm), sex, and presence and number of external *L. panopaei* parasite reproductive structures, called externae. While counting only external signs of parasitism produces a conservative estimate of prevalence, this is the standard approach used in this system (see references in Table S1.2). Additional methodological details are given in Appendix S1.

Statistical analysis for field sampling followed a hierarchical or stratified design. At the highest level, there are three regions that differ in their history of parasitism, where the parasite has a status of native, introduced, or absent. Within each of these regions, three different estuaries were sampled, and within each estuary, collectors were placed at three to four distinct sites. Finally, each of these sites is represented by one to two distinct samples, each of which represents a different time point (summer and fall). Prevalence was first calculated at the smallest scale for each site × time sample (Table 1; Rothman, 2002), as the number of visibly infected crabs divided by the total number of crabs above the minimum size for visible infection (3.9 mm CW for *R. harrisii*; 5.8 mm CW for *E. depressus*; see Appendix S1 for further detail).

**Table 1:** Summary of field survey data giving species and infection status by site and time

Region estuary	Site	Summer					Fall				
		*N* CCUs	*Rhithropanopeus harrisii*	*Eurypanopeus depressus*	*Sal.*	*Temp.*	*N* CCUs	*Rhithropanopeus harrisii*	*Eurypanopeus depressus*	*Sal.*	*Temp.*
Native											
Louisiana	LA2	2	97 (0/54)	0	8.6	31.8 ± 1.4	2	**287 (2/235)**	0	9.0	27.5 ± 2.8
	LA3	2	7 (0/5)	1 (0/1)	9.8		2	24 (0/21)	1 (0/1)	11.6	
	LA4	1	**14 (1/10)**	3 (0/3)	5.3		2	119 (0/58)	1 (0/1)	13.5	
	LA5	0	–	–	–		2	76 (0/59)	0	5.1	
Florida—Gulf	AP1	2	206 (0/118)	20 (0/20)	11.1	30.7 ± 1.3	2	78 (0/56)	**115 (1/15)**	18.3	27.0 ± 2.1
	AP2	2	194 (0/124)	38 (0/32)	13.5		2	17 (0/1)	**279 (1/110)**	21.1	
	AP3	2	0	**67 (2/49)**	31.5		0	–	–	‐	
	AP4	0	–	–	–		2	14 (0/4)	89 (0/42)	17.5	
Florida—Atlantic	FP1	2	41 (0/40)	16 (0/10)	11.0	30.9 ± 1.3	2	16 (0/12)	14 (0/13)	11.4	29.2 ± 1.0
	FP2	2	0	22 (0/14)	14.5		0	–	–	‐	
	FP3	2	159 (0/107)	58 (0/44)	8.3		2	63 (0/55)	6 (0/4)	15.0	
	FP4	0	–	–	–		2	148 (0/97)	17 (0/14)	5.8	
Total native			**718 (1/458)**	**225 (2/173)**				**842 (2/598)**	**522 (2/200)**		
Introduced											
Florida—Atlantic	ML1	2	**9 (4/9)**	2 (0/2)	21.6		0	–	–	‐	
	ML2	2	**120 (9/29)**	**2 (1/2)**	14.2		2	**99 (8/69)**	0	13.2*	25.3 ± 1.5
	ML3	2	**38 (10/37)**	0	12.4	30.6 ± 1.9	2	32 (0/16)	0	0.3*	
	ML5	0	–	–	–		2	4 (0/3)	**4 (1/4)**	19.0*	
	ML6	0	–	–	–		2	**49 (5/38)**	0	1.0*	
South Carolina	SC1	2	**206 (9/84)**	3 (0/3)	7.5		1	**29 (1/12)** *	1 (0/1)*	0.2*	
	SC2	2	**180 (2/43)**	7 (0/6)	10.8	30.7 ± 0.6	2	28 (0/8)*	4 (0/3)*	2.1*	23.7 ± 2.4
	SC3	2	55 (0/9)	11 (0/9)	20.8		0	–	–	‐	
	SC4	0	–	–	–		2	70 (0/40)*	0*	0.1*	
Maryland	MD1	2	**43 (36/42)**	**19 (6/17)**	10.9		2	**16 (14/16)**	**10 (1/7)**	14.5	
	MD2	2	**53 (9/43)**	0	11.9		2	**51 (27/51)**	0	14.3	
	MD3	1	359 (0/318)	0	9.4		1	**87 (10/84)**	0	16.5	
Total introduced			**1,063 (79/614)**	**44 (7/39)**				**465 (65/337)**	**19 (2/15)**		
Absent											
New Jersey	NJ1	2	130 (0/118)	0	14.5	27.2 ± 1.5	2	61 (0/54)	0	8.0	13.7 ± 1.7
	NJ2	2	23 (0/13)	21 (0/13)	18.8		0	–	–	‐	
	NJ3	2	96 (0/76)	4 (0/2)	13.1		2	88 (0/82)	5 (0/5)	9.6	
Massachusetts	MA1	2	0	95 (0/86)	25.3		0	–	–	‐	
	MA2	1	5 (0/5)	0	1.1		0	–	–	‐	
	MA3	2	22 (0/22)	0	11.3	25.6 ± 1.9	0	–	–	‐	
New Hampshire	NH1	2	301 (0/152)	25 (0/13)	27.9		0	–	–	‐	
	NH2	2	164 (0/120)	5 (0/3)	27.0	25.2 ± 1.2	0	–	–	‐	
	NH3	2	54 (0/43)	0	21.3		0	–	–	‐	
Total absent			795 (0/549)	150 (0/117)				149 (0/136)	5 (0/5)		
Grand total			2,576	419				1,456	546		

Note

Count is total number of crabs found; number in parentheses is the number of visibly infected crabs over the number of crabs above the size threshold for visible infection (*R. harrisii*: 3.9 mm; *E. depressus*: 5.8 mm). Samples in which the parasite was found are indicated in bold. CCU, Crab Collector Unit; Sal., salinity in PSU, as a point measurement at time of sampling; Temp., temperature in °C, averaged over the prior 30 days.

* Sampling occurred just after a major rain/flooding event: Salinity is likely abnormally low, and samples may also be affected.

At broader spatial and temporal scales, two approaches were used to describe parasitism. First, the extent of parasitism in an estuary or region was calculated as the proportion of samples in which *L. panopaei* was found, giving a rough idea of how widespread the parasite was within that area. For this, we used a binomial model based on parasite presence or absence in a given sample, with site nested in estuary as a random effect; the southeastern Florida estuary (FP) was excluded since we could not confirm the presence of *L. panopaei* here. Second, prevalence within each estuary or region was calculated only for samples where the parasite had been found (Table 2).

**Table 2:** Parasite prevalence by estuary and regional parasite status (native or introduced); for introduced regions, approximate date of introduction is given in parentheses

Region	Estuary	*N* samples	*N* para	Prop para	Within parasitized samples only		
					*N* crabs	Overall prevalence	Range of prevalence
Native	Louisiana	7	2	28.6	245	1.2	0.9–10.0
Native	Florida—Gulf	5	0	0	0	0	–
Native	Florida—Atlantic	5	0	0	0	0	–
Overall native		12*	2	16.7	245	1.2	0.9–10.0
Introduced (2005)	Florida—Atlantic	7	5	71.4	182	19.8	11.6–44.4
Introduced (c. 1993)	South Carolina	6	3	50.0	139	8.6	4.7–10.7
Introduced (1964)	Maryland	6	5	83.3	236	40.7	11.9–87.5
Overall introduced		19	13	68.4	557	25.9	4.7–87.5

Note

*N* samples = number of site × time samples within each estuary; *N* para = number of site × time samples where *Loxothylacus panopaei* was found; Prop para = proportion of samples where *L. panopaei* was found. Prevalence was calculated only for those samples where *L. panopaei* was present. Overall prevalence was calculated across all adult crabs in a given region; range of prevalence indicates the range of prevalence values calculated for each sample within a given region. *N* crabs, number of crabs in each sample above the minimum size for visible infection; prevalence, proportion of crabs infected.

* Excluding the Atlantic Florida estuary, where the presence of *L. panopaei* could not be confirmed.

We tested whether regional parasite status influenced individual infection probability using a generalized linear mixed model (binomial with logit link function), with host size as a covariate and a nested site:estuary random effect to control for multiple sampling. Host sex did not improve the model fit and was excluded (*p* > .5). Modeling tests were carried out separately for *R. harrisii* and *E. depressus* using all individuals sampled in the parasite's native and introduced ranges with the “lme4” package in R v3.3.2.

### 2.3 Literature survey

Data on *L. panopaei* prevalence were extracted from published literature and from four unpublished data sets including empirical surveys conducted for this study (Table S1.2, Appendix S2). In order to be included, data had to meet the following criteria: Sampled sites were in the native range of *R. harrisii* or *E. depressus,* crabs had been examined for *L. panopaei* infection after collection (i.e., crabs were not specifically selected for infection status during sampling), prevalence rates were reported by crab species, and number of crabs examined was provided. For records of absence, the study had to explicitly state that *L. panopaei* was searched for and not found despite the presence of viable hosts. We used prevalence rates either reported in the literature or, when possible, calculated directly from reported numbers of infected and uninfected crabs. Several studies spanned both native and introduced regions of the parasite's range. Finally, we only included records where prevalence was based on samples of ≥10 individual crabs. Additional details can be found in Appendix S1.

For the meta‐analysis data, we calculated the mean prevalence in each region and its 95% confidence interval using the Freeman–Tukey double arcsine transformation (Freeman & Tukey, 1950), back‐transformed to proportions per Miller (1978) using an unweighted mean and implemented in the R package “metafor” (Viechtbauer, 2010). This approach was chosen in part because it handles proportions equal to zero well, and our data included multiple samples in which the parasite was not found. We calculated mean prevalence both with and without samples in which the parasite was not found.

To compare both presence and prevalence of the parasite between its native and introduced ranges, we used linear mixed models in the R package “lme4.” We first compared the proportion of sites where *L. panopaei* was found between the parasite's native and introduced ranges using a binomial model based on parasite presence or absence, with site nested in estuary as a random effect. We tested parasite prevalence against regional parasite status and status‐host species interaction as fixed effects, with site nested in estuary and reference (e.g., study from which the data derived) as random effects. For all meta‐analysis models, we calculated the theoretical marginal and conditional *R* ^2^ using the approach of Nakagawa and Schielzeth (2013) implemented in the R package “MuMIn” (Bartoń, 2019).

### 2.4 Experimental infection

A subset of crabs 4–8 mm CW with no visible externae were collected live and experimentally exposed to *L. panopaei* in the laboratory to test susceptibility to parasitism under controlled conditions. Crabs for this experiment derived from six estuaries in total, two from each region (naïve: NH, NJ; short‐term interaction: SC, ML; long‐term interaction: AP, LA; Figure 1a; Table 3). In the laboratory, all crabs were held individually in 50 ml of 15 PSU artificial seawater at 20°C and a 12‐hr:12‐hr light:dark cycle, conditions shown to be within the optimal range for both host and parasite (Reisser & Forward, 1991; Walker & Clare, 1994). Crabs were fed a diet of commercial crab food (Hikari Crab Cuisine), with full water exchanges every other day, and were monitored daily for molting and mortality. Crabs showing visible signs of *L. panopaei* infection after their first laboratory molt (a result of internal infection contracted in the field) were removed from the experiment.

**Table 3:** *Rhithropanopeus harrisii* susceptibility to parasitism by *Loxothylacus panopaei*, as the percentage of hosts becoming infected after a single exposure to the parasite

Region	Estuary	Site	Parasitized	Unparasitized	Total	Susceptibility (%)
Native	Louisiana	LA2	4	9	13	30.8
	Florida—Gulf	AP1	8	23	31	25.8
Overall native			12	32	44	27.3
Introduced	Florida—Atlantic	ML2	4	13	17	23.5
	South Carolina	SC1	4	16	20	20.0
Overall introduced			8	29	37	21.6
Absent	New Jersey	NJ1	16	10	16	62.5
	New Hampshire	NH2	6	8	14	42.9
Overall absent			16	14	30	53.3

Note

Site is the specific sampling site where experimental crabs were collected, as in Table 1. Parasitized, unparasitized, and total are numbers of crabs in each category.

Within 24 hr after molting in the laboratory, crabs were exposed to 100+ competent parasite cyprids per Alvarez, Hines, and Reaka‐Kudla (1995). Crabs are only susceptible to parasitism by *L. panopaei* cyprid larvae shortly after molting; in parasitized adult crabs, a virgin externa (visible with the naked eye) typically emerges at the next molt after infection (Alvarez et al., 1995). Parasite larvae were obtained from *R. harrisii* collected in the Chesapeake Bay that had mature externae. These were held in the laboratory under the same conditions as experimental crabs; upon hatching, nauplius larvae were reared to the infective cyprid stage (2 days) before being used for experimental infections. Each crab was exposed to a mix of competent larvae derived from two different parasite individuals. Crabs and cyprids were held together under experimental conditions for 24 hr, and then water was fully exchanged to remove all remaining larvae.

Exposed crabs were maintained under laboratory conditions through their next molt following exposure, after which they were checked for virgin parasite externae to determine whether or not they had been parasitized. The few exposed crabs which did not molt a second time during the experiment were tested for the presence of *L. panopaei* DNA in their body cavities using the species‐specific Lxpa‐L and ‐R primers designed by Kruse and Hare (2007).

To test for differences in susceptibility among laboratory‐exposed crabs, we used a binomial generalized linear mixed model implemented in the R package “lme4,” with significance assessed using the Type II sum of squares test in the “ANOVA” function from the R package “car” (Fox & Weisberg, 2011). This model used laboratory infection status (parasitized or unparasitized) as the response, with interaction history and crab size as fixed effects and site as a random effect. Crab sex and interactions did not improve the model and were not included.

All plotting used the “ggplot2” package in R (Wickham, 2009).

## 3 RESULTS

### 3.1 Prevalence in field survey

In total, we sampled 5,088 panopeid crabs, primarily *R. harrisii* (79.2%) and *E. depressus* (19.0%). The remainder of the samples were comprised of *Panopeus herbstii* (1.2%) and *Dyspanopeus sayi* (0.6%). We found evidence of *L. panopaei* parasitism in *R. harrisii* in five of nine surveyed estuaries and in *E. depressus* at three of nine estuaries. Infection rate in *R. harrisii* was highly variable across regions with different histories of parasitism. As expected, we found no evidence of the parasite in our putatively parasite‐absent estuaries in New Jersey, Massachusetts, and New Hampshire (0/12 samples; *N* = 944 crabs). Additionally, we found no evidence of *L. panopaei* (ER clade) in our putatively parasite‐native estuary in southeastern Florida. In a second native estuary, in Gulf coast Florida, we saw no *L. panopaei* infections in *R. harrisii* but did encounter the parasite in *E. depressus*, confirming its presence in the estuary. Because of this, we retained Gulf Florida as a parasite‐native estuary but conducted relevant tests without including the southeastern Florida estuary in the parasite‐native region as noted.

In *L. panopaei*'s native range, 16.7% (2/12; *N* = 1,133 crabs) of *R. harrisii* samples contained *L. panopaei*, while in the parasite's introduced range it was encountered significantly more frequently at 68.4% (13/19; *N* = 1,528 crabs) of samples (*z* = −2.59; *p* = .0095; Table 1). Among parasitized samples, overall prevalence in the native‐parasite region averaged 1.2% (range: 0.9%–10.0%), while in the introduced‐parasite region, prevalence was substantially higher at 25.9% (range: 4.7%–87.5% Figure 1b; Table 2). Across all *R. harrisii* adults surveyed in the parasite's native and introduced ranges, linear mixed models found that crabs in the parasite's introduced range were significantly more likely to be parasitized than those in its native range (*z* = −4.45, *p* < .001).

Although we found far fewer *E. depressus* than *R. harrisii* due to our focus on brackish areas, we observed a similar pattern of infection prevalence in this host species. In *L. panopaei's* native range, 30% of sampling events detected *L. panopaei* (3/10; *N* = 614 crabs), in comparison with 40% percent of events within its introduced range (4/10; *N* = 63 crabs; *z* = −0.65, *p* = .52). Among sampling events with *L. panopaei,* prevalence was higher in the introduced range: 25.7% infected (range: 14.3%–50.0%) versus 1.3% (range: 0.1%–6.7%) infected in *L. panopaei*'s native range. Modeling found that the probability of parasitization in *E. depressus* was significantly higher where the parasite was introduced relative to its native range (*z* = −2.41, *p* = .016).

### 3.2 Prevalence in the literature

**Figure 2:**
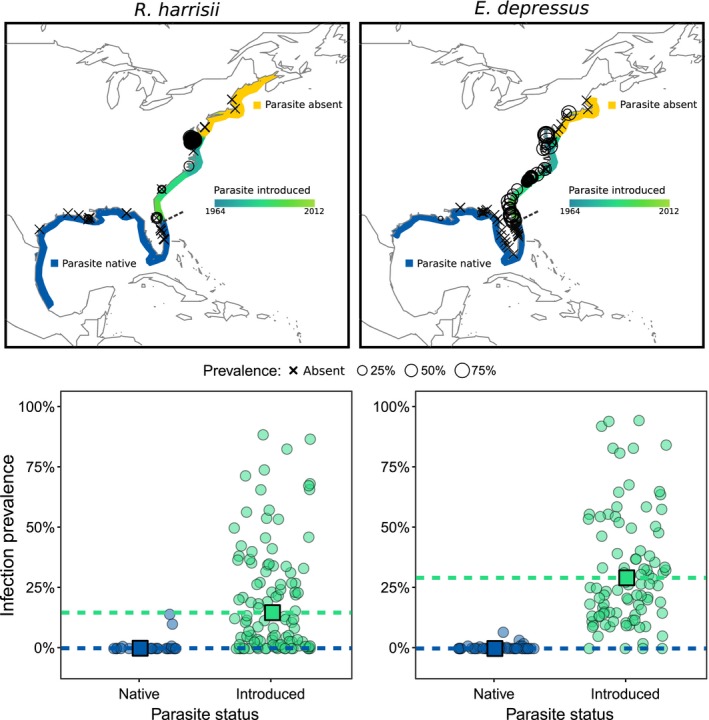
Prevalence of *Loxothylacus panopaei* in primary hosts *Rhithropanopeus harrisii* (left panels) and *Eurypanopeus depressus* (right panels) based on data from the published literature, this study, and unpublished data (Table S1.2). Top: Prevalence by geography, with the size of the circle scaled to prevalence. The dashed line indicates Cape Canaveral. Bottom: Comparison of prevalence data in the native and introduced range of *L. panopaei*. Circles indicate single prevalence estimates; square points and dashed lines give the mean prevalence across sites/studies in each region. Points are jittered horizontally for clarity

For *R. harrisii*, *L. panopaei* was present in 84.3% of introduced range records (91/108; Figure 2), significantly more frequently than the 16.7% of native range records where the parasite was encountered (4/24; *z* = −3.31, *p* < .001). Average prevalence, including records where the parasite was not found, was also significantly higher in the introduced range, at 14.7% (CI: 13.9%–15.5%), than in the native range at 0.1% (CI: 0%–0.7%; *z* = −2.92, *p* = .0035; marginal *R* ^2^ = 0.35, conditional *R* ^2^ = 0.79). If prevalence is averaged only over records where the parasite was found, this difference remains significant at 19.8% (CI: 18.8%–20.8%) prevalence in the introduced range versus 4.5% (CI: 1.4%–9.0%) in the native range (*z* = −2.38, *p* = .017).

In *E. depressus*, the difference in parasite presence was even more pronounced: 95.7% of records in the introduced range reported the parasite (90/94), as opposed to 20.8% in the native range (10/48; *z* = −3.62, *p* < .001). Overall prevalence was 29.0% (CI: 27.3%–30.7%) in the introduced range, significantly higher than the 0.03% (CI: 0%–0.3%) in the native range, when averaged over all records (*z* = −9.43, *p* < .001; marginal *R* ^2^ = 0.55, conditional *R* ^2^ = 0.77). Again, this remains significant when unparasitized records are excluded: 31.0% (CI: 29.3%–32.8%) prevalence in the introduced range as opposed to 1.4% (CI: 0.6%–2.5%) in the native range (*z* = −6.40, *p* < .001).

Models indicated that both presence and prevalence were significantly higher in the parasite's introduced range for both species (presence: *z* = −4.04, *p* < .001; prevalence: *z* = −9.37, *p* < .001; marginal *R* ^2^ = 0.50, conditional *R* ^2^ = 0.75). Presence was similar across both host species, in both the parasite's native and introduced ranges (introduced: *z* = −1.85, *p* = .064; native: *z* = 0.745, *p* = .46). By contrast, parasite prevalence was significantly higher in *E. depressus* than in *R. harrisii* (*z* = −3.54, *p* < .001) in the parasite's introduced range, though there was no difference in prevalence between host species in the parasite's native range (*z* = 0.38, *p* = .70). When considering only sites where the parasite was found, prevalence remained significantly higher in the introduced than native range (*z* = −6.88, *p* < .001), but was similar across both host species, in both the parasite's native and introduced ranges (introduced: *z* = −1.86, *p* = .063; native: *z* = 1.58, *p* = .11).

### 3.3 Susceptibility in the laboratory

We followed 111 crabs in the laboratory from experimental parasite exposure through determination of infection status. While there was some mortality during the 6‐month experimental duration, it was unrelated to interaction history (the authors, unpublished data). We obtained susceptibility data from 16–31 crabs per site: 30 crabs in total from the two sites in the range where the parasite was absent, 37 crabs from the two sites where the parasite was introduced, and 44 crabs from the two sites where the parasite was native. Overall, 32.4% of crabs became parasitized after this single laboratory exposure (36/111).

Within the experimental range of 4–8 mm CW, size did not significantly affect a crab's chances of being parasitized (*χ* ^2^ = 1.52; *p* = .22). By contrast, interaction history did significantly change a crab's susceptibility (*χ* ^2^ = 8.99; *p* = .011). In naïve crabs, 53.3% (16/30) were parasitized, a significantly elevated susceptibility compared with 27.3% (12/44) of crabs from the parasite's native range (*z* = 2.39; *p* = .045) and 21.6% (8/37) of crabs from the parasite's introduced range (*z* = 2.794; *p* = .014; Figure 1c; Table 3). There was no difference in susceptibility between crabs from the parasite's native range and those from the parasite's introduced range (*z* = −0.69; *p* = .77).

## 4 DISCUSSION

Host–parasite evolutionary dynamics are increasingly altered by anthropogenic global change and increased vector traffic, as hosts and parasites are introduced beyond their natural boundaries. Several studies have illustrated significant geographic variation in parasitism where evolutionarily naïve populations are disproportionately affected by introduced parasites. For instance, native Hawaiian stream fishes experienced much higher prevalence of an introduced parasitic nematode compared with introduced fishes from the parasite's native range (Gagne, Heins, McIntyre, Gilliam, & Blum, 2016). Similarly, the collapse of native mud shrimp populations in western North American has been linked to the introduction of a parasitic bopyrid isopod, which is far more prevalent there than in its native Asian waters (Hong, Lee, & Min, 2015). Parasite virulence may also change with invasion: A recent review concluded that 85% of studies (14/16) found higher virulence of introduced parasites in novel native hosts than in the coevolved hosts with which they invaded (Lymbery et al., 2014). Moreover, parasites and pathogens transferred with introduced hosts can have significant negative impacts on populations and communities of native species in the recipient region, including the local extinction or extirpation of native hosts (Shields et al., 2015; Strauss, White, & Boots, 2012).

However, previous work differs from this study since our results do not represent host‐switching by the parasite. Instead, molecular analyses suggest that a single *L. panopaei* lineage (the ER clade, which primarily infects *R. harrisii* and *E. depressus*) was probably introduced from the Gulf of Mexico to the Chesapeake Bay (Kruse et al., 2011). This biogeographic mismatch between widespread hosts and a historically more geographically restricted parasite sets up the relatively unexplored dynamic of a parasite introduction to naïve hosts without host‐switching. There are few studies of this phenomenon in macroparasite systems (but see Feis, Goedknegt, Thieltges, Buschbaum, & Wegner, 2016). Most comparable examples to this system come from the medical literature, where emerging microbial pathogens are introduced to naïve populations of widespread hosts such as humans or agricultural species (Fenner, 1993; Schrag & Wiener, 1995). However, microbial pathogens evolve and spread much more rapidly than metazoan parasites such as *L. panopaei*, in which a single parasite typically infects a single crab over the host's lifetime.

### 4.1 Prevalence and susceptibility

Here, we found a significant increase in the prevalence of an introduced castrating parasite relative to its prevalence in the same host species in its native range. Increased prevalence after invasion is supported by both a controlled empirical survey and a meta‐analysis of historical prevalence levels (Figures 1 and 2). This finding provides initial support for our hypothesis that the host may have evolved to resist parasitism where it shares a long‐term history with its parasitic castrator (Kruse & Hare, 2007). Hosts and parasites can act as powerful selective agents on one another, and theory predicts that host and parasite may continually coevolve in response to these pressures (e.g., the coevolutionary arms race; Dawkins & Krebs, 1979; Tellier, Moreno‐Gámez, & Stephan, 2014; Thompson, 1999). Under this framework, mud crab hosts in the Gulf of Mexico may have evolved to avoid or resist *L. panopaei* parasitism over millennia of coexistence. By contrast, mud crabs in the Chesapeake Bay and other parts of the parasite's introduced range were naïve to rhizocephalans and thus may have lacked evolved defenses to this recently arrived parasite, which has a finely honed ability to encounter and infect these host species.

Population genomics of the nine populations in this study have identified extensive divergence between *R. harrisii* in the Gulf Coast and those in the Chesapeake Bay, suggesting that *L. panopaei* has truly become established in a naïve host population rather than being introduced along with a coevolved host population (the authors, in review). An earlier experimental study in the parasite's introduced Chesapeake Bay range suggested that susceptibility to parasitism was not strongly heritable within 12 families of mud crabs, but this initial experiment within a single population did not compare evolved differences between host populations from the parasite's native range and previously naïve host populations (Grosholz & Ruiz, 1995). Our susceptibility data support a role for evolutionary change in influencing prevalence, by demonstrating that naïve crabs are significantly more susceptible to infection under controlled exposures than are crabs from populations where the parasite is native (Figure 1c).

Notably, we found no difference in susceptibility between crabs from the parasite's native and introduced ranges despite marked differences in field prevalence. We suspect that our experimental design has contributed to (or perhaps caused altogether) the unexpectedly low susceptibility in crabs where the parasite has invaded. Our experimental design relied on uninfected crabs collected as adults from the field, so we inadvertently selected for individuals that had repeatedly escaped or resisted parasitism prior to collection. *Rhithropanopeus harrisii* can be parasitized as early as the megalopal stage, and by the time crabs reach our minimum experimental size of 4 mm CW they have gone through approximately five molts during which they are vulnerable to parasitism (Alvarez et al., 1995). While the likelihood of a vulnerable crab encountering a competent parasite was probably low in the parasite's native range, where externae prevalence was <5%, it was no doubt considerably higher where the parasite was introduced and significantly more prevalent (Figure 1b). We suggest that the low susceptibility in crabs from the parasite's introduced range (similar to that observed in the native range) may be due to parasitism in the field selectively removing susceptible crabs from our experimental pool. Alternatively, or additionally, it is possible that crab populations in the introduced range have rapidly evolved lower susceptibility in response to parasite pressure over multiple generations of selective pressure. Two notable examples in nature include a cricket population which evolved a distinct “silent calling” morphology within 20 generations of the introduction of a parasitoid attracted by sound (Zuk et al., 2006), and trout populations which evolved increased juvenile resistance to whirling disease within 10 years of its introduction (Miller & Vincent, 2008).

By contrast, in the parasite's native range, millennia of interaction between host and parasite may have acted to reduce the host populations' overall susceptibility to the parasite. While these data are preliminary, the significant increase in susceptibility in naïve populations relative to populations in which the parasite is native suggests that parasite‐induced selection may have shaped the observed patterns. Given the low prevalence of the parasite in its native range, both in our study and in every prior study of which we are aware (Figures 1 and 2), it seems unlikely that our coevolved populations face the same risk of infection per generation (and thus experimental confounding) as do their counterparts where the parasite is introduced. Future laboratory experiments examining susceptibility in crabs raised from larvae in parasite‐free, controlled laboratory environments (preferably over multiple generations) would be instrumental in elucidating the true role of long‐term evolution in shaping observed patterns of susceptibility.

We note also that our work examines only half of the potential coevolutionary story. While we focus here on host evolution in response to parasitism, it is likely that the parasite is also evolving as it expands into new host populations (Kelehear, Brown, & Shine, 2012). Interestingly, our findings of increased susceptibility in novel host populations contrasts with work done in a snail host–trematode parasite system supporting the matching alleles hypothesis, in which hosts are more susceptible to coevolved parasite populations (King, Delph, Jokela, & Lively, 2009). In one study, clonal snail lines that had escaped parasitism via invasion were largely susceptible to parasites from their native range and resistant to parasites from outside of that native range (Fromme & Dybdahl, 2006). Work on the macroparasite *Mytilicola intestinalis*, which has invaded naïve mussel populations, has shown the rapid development of distinct host–parasite relationships at separate edges of the invasion front (Feis et al., 2016). Transcriptomic work reinforced this result, showing that gene regulation in both infected hosts and infecting parasites differed depending on whether specific host and parasite populations were sympatric or allopatric (Feis, John, Lokmer, Luttikhuizen, & Wegner, 2018).

We conducted susceptibility experiments using a single parasite population from Chesapeake Bay, which was allopatric to all host populations. To examine potential evolution of the parasite and its role in shaping prevalence and susceptibility, we suggest future experiments to compare susceptibility between sympatric and allopatric host–parasite population pairs, including sites where the parasite is native and invasive. In addition, in this system, the parasite is potentially coevolving with two host species that differ in their environmental tolerances (Kruse & Hare, 2007; Williams, 1984), presenting opportunities to test potential coevolution when the parasite is not limited to a single host species. While we present the susceptibility data in this paper as initial evidence of potential coevolution in this system, there is much more work to be done.

### 4.2 Prevalence, environment, and evolutionary dynamics

The observed increase in prevalence after introduction may also be influenced by environmental or ecological differences across sites. Temperature in particular is a strong selective force that influences the survival and transmission of many parasites (Auld & Brand, 2017; Harvell et al., 2002), and there is evidence that elevated temperatures may disadvantage *L. panopaei* reproduction in its introduced range (Gehman, Hall, & Byers, 2018). While we cannot yet fully disentangle temperature and latitude from invasion history in this system, we suggest that the pattern of prevalence does not support either factor as a driving force. In our empirical study, three estuaries are very similar in latitude (LA: 29.125°N; ML and AP: 29.625°N) and number of hot days (days with SST ≥25°C; ML: 163; AP: 165; LA: 165). Despite this broad environmental similarity, the northeastern Florida site (ML) where the parasite is introduced has a markedly higher parasite prevalence than its “sister” sites in the parasite's native range (LA and AP; Figure 1b).

Smaller scale environmental differences may also influence parasite prevalence patterns, as has been observed for other Rhizocephalan parasites (Sloan, Anderson, & Pernet, 2010). We recorded highly variable parasite prevalences both among and within estuaries, most notably in Maryland where prevalence ranged from 0% to 87.5% (Table 1). This wide range of prevalence may be due in part to salinity differences between sites; published data show strong salinity dependence of *L. panopaei* larval development. In laboratory studies, larval development has shown a sharp decline at around 10 PSU, with minimal development success below that threshold (Reisser & Forward, 1991; Walker & Clare, 1994). In a field study on a sister lineage of *L. panopaei* (clade P), a salinity threshold at ~15 PSU was observed in seasonal collections of host *Panopeus obesus*, as environmental salinity changed with rainfall (Tolley et al., 2006).

A low‐salinity barrier to parasite development may contribute to the observed difference in parasite prevalence between *R. harrisii* and *E. depressus* in our literature review. Average prevalence in *E. depressus* was 22 times higher in the introduced than native ranges, in contrast to a fourfold increase in *R. harrisii,* for samples where the parasite was present (Figure 2). This difference may reflect the ability of the mesohaline *R. harrisii* to exploit a low‐salinity refuge where *L. panopaei* cannot consistently develop, whereas the more polyhaline *E. depressus* has little environmental respite from parasitism (Williams, 1984). A recent analysis did not find salinity to be a significant factor in rates of *E. depressus* parasitism, but only included sites where salinity was 30–37 PSU, well above the potential refuge threshold of 10 PSU (Gehman et al., 2016). More generally, low‐salinity refugia from parasitism appear to be a relatively widespread phenomenon for estuarine species including other crabs (Dunn & Young, 2013; Ford, Scarpa, & Bushek, 2012).

### 4.3 Coevolution and biological introductions

Species introductions of hosts, both with and without their parasites, also offer valuable opportunities to explore the potential role of coevolution in shaping host–parasite interactions. In a different Rhizocephalan system, the crab host *Charybdis longicollis* initially escaped its parasite *Heterosaccus dollfusi* as it spread through the Suez Canal into the Mediterranean Sea (Galil & Lützen, 1995). In this system, the parasite eventually caught up, infecting introduced host populations after 30 years of separation (Innocenti & Galil, 2007). Parasite prevalence quickly climbed in the introduced region, though it is unclear how these levels compare to prevalence in both species' native range (Innocenti & Galil, 2007). This may be consistent with modeling work suggesting that lag times between host and parasite range expansion could substantially affect the evolutionary trajectory of hosts, potentially leading to increased susceptibility in hosts that have (temporarily) shed their parasites (Phillips et al., 2010).

While *R. harrisii* has been introduced to many global regions, in all of these cases it appears that *L. panopaei* has not yet followed it (Fofonoff, Ruiz, Steves, Simkanin, & Carlton, 2019; Fowler, Forsström, von Numers, & Vesakosk, 2013). Understanding the source region of current (and future) introductions of hosts and their interaction history with potential parasite introductions may have important implications for the ecology and dynamics of introduced crab populations and their ecosystems. For example, on the west coast of North America and Europe, it appears that *R. harrisii* introductions have derived from naïve native crab populations before the invasion and spread of *L. panopaei* (Forsström, Ahmad, & Vasemägi, 2017; Petersen, 2006; Projecto‐Garcia, Cabral, & Schubart, 2010; J. A. Darling, personal communication). Given the increased susceptibility we observed in naïve host populations relative to populations with long histories of interaction with the parasite, this suggests that many introduced populations may share an increased ancestral susceptibility to *L. panopaei* should it spread more globally. Thus, the ancestral source(s) may affect the likelihood of future parasite establishment, since inoculation with the same number of parasite propagules is more likely to infect susceptible hosts, controlling for other factors. This, in turn, has potential implications for the population dynamics of the host and its downstream effects on the invaded community.

In contrast, *R. harrisii* in the Gulf of Mexico with extensive historical interaction with *L. panopaei* have a significantly lower susceptibility to *L. panopaei* infection than naïve crabs, as demonstrated here. Populations that evolved with the parasite and then escaped it may permit a test of the time scale on which evolved resistance is retained; both theoretical and empirical work have suggested that resistance may be lost over time if the host is removed from the selective pressure imposed by its parasite (Phillips et al., 2010; Keogh, Miura, Nishimura, & Byers, 2017). Thus, the existence of multiple host introductions from multiple sources, with a variety of histories of parasitism, provides a uniquely promising system in which to explore the potential influence of host–parasite coevolution on biological introductions.

Biological introductions can transport both visible species and their symbiotic biological communities. In the marine realm, concern about parasite introductions has been limited and concentrated primarily on aquaculture species such as salmon and oysters (Vignon & Sasal, 2010). Part of this issue lies in a dearth of knowledge of marine parasite communities, and also relatively limited effort to detect introductions of parasites and small organisms in general, making it difficult to predict potential parasite co‐introductions or even to detect them (Goedknegt et al., 2016; Ruiz et al., 2000). Our study highlights an important example of marine parasite introduction and an unusual case of parasite introduction without host‐switching. This kind of invasion may be increasingly important but underreported in marine communities, where species can live across wide spatial and environmental gradients. However, the evolutionary consequences of such introductions may be severe. As species introductions continue and the environment changes in many dimensions, parasite introductions may have an increasing influence on the ecology and evolution of native species and communities.

## CONFLICT OF INTEREST

The authors declare that they have no conflict of interests regarding this publication.

## Data Availability

All field survey data are publicly available via figshare at https://doi.org/10.6084/m9.figshare.9736532. Published literature survey data are publicly available via figshare at https://doi.org/10.6084/m9.figshare.9750605. Susceptibility data are part of a larger data set which will be added to figshare upon publication of the relevant manuscript; before that point, the data used in this study will be made available upon request.
