# Supplementary material for: Recent introductions reveal differential susceptibility to parasitism across an evolutionary mosaic
Source: Evol Appl. 2019 Sep 25;13(3):545–58. doi: 10.1111/eva.12865 (PMC7045710; doi:10.1111/eva.12865)
Supplement: Supplementary file 1 [file EVA-13-545-s001.doc]

**Recent introductions reveal differential susceptibility to parasitism**

**across an evolutionary mosaic**

**APPENDIX S1: Additional Methodological Details**

***Field sampling***

Sampling was carried out in nine estuaries located across the native range of *Rhithropanopeus harrisii*. Estuaries were chosen based on geography, freshwater inflow, and (when available) reported presence of *R. harrisii* and/or *Loxothylacus panopaei*. We sampled 33 sites, 3-5 per estuary (Tables 1, S1.1). Sixteen sites were sampled twice, and 17 sites were sampled once. With the exception of sites in New Jersey, Massachusetts, and New Hampshire, single-sampled sites were a result of moving collectors to new sites after the first sampling if *R. harrisii* catch was poor (e.g., few or no mud crabs were caught, or the mud crabs caught were not *R. harrisii*).

Trapping was carried out using “crab collectors”, unbaited 19 x 22 x 15.75 cm plastic crates (United Solutions, Leominster, MA) filled with autoclaved oyster shell and topped with 1.9 cm plastic mesh (Tenax, Baltimore, MD) affixed with zip ties. Collectors were deployed on the benthos attached to rope connected to shore for easy retrieval. Collectors were deployed in the early summer, proceeding roughly from south to north to account for latitudinal differences in crab abundance and activity and parasite emergence. At each check, collectors were emptied into a sieve with 2 mm mesh. All panopaeid crabs ≥ 2mm carapace width (CW) were collected (live or in 95% ethanol) for later sorting in the laboratory. Collectors and oysters were thoroughly checked twice for panopaeids, and fine forceps were used to extract crabs from tight spaces and crevices.

In the laboratory, all collected panopaeids were examined under a dissecting microscope and the following data were collected: species, maximum carapace width (mm), sex, and presence and number of *L. panopaei* parasites. For species identification, Williams (1984) was used as a primary guide. All crabs with clear signs of infection were classed as infected, based on the presence of parasite reproductive sacs, or externae, and scars from prior externae. This is a conservative estimate of infection, given that *L. panopaei* infection is not visible externally in its early stages. However, visible infection appears to be a good predictor of relative rates of latent infection. As part of a different study, crabs from each of our studied estuaries were maintained in the laboratory over multiple molts and showed latent infection rates of 0% for parasite-absent estuaries, 0-2% for parasite-native estuaries, and 3-38% for parasite-introduced estuaries (the authors, unpublished data).

Environmental data were collected at each site when traps were both deployed and retrieved. Salinity and temperature were measured with a handheld YSI Pro30 meter (YSI, Yellow Springs, OH) at the time of sampling.

***Meta-analysis***

For unpublished data, most of which we collected in the course of other studies, we calculated prevalence for *R. harrisii* >3.9 mm CW. We chose this cutoff because external signs of infection do not become visible until a crab reaches a minimum size threshold. Although infection can take place as early as the megalopal stage, the minimum size at first emergence is given as 4 mm CW (Alvarez, Hines, Reaka-Kudla, 1995); in our data, the smallest *R. harrisii* individual with a visible infection was 3.94 mm CW. We used a minimum size threshold of 5.8 mm CW to calculate prevalence in *Eurypanopeus depressus*; this represents the smallest size reported in the literature for visible infection in this species (O'Shaughnessy, Freshwater, Burge, 2014). Most published studies relied on hand-collecting from intertidal and shallow subtidal environments, and are likely to have excluded juvenile crabs. Several such studies explicitly included only crabs ≥5 mm CW, and in those without a minimum cutoff which reported size distributions, few crabs <5 mm CW were reported (Table S1.2).

Records of sampling in the parasite's introduced range were only included if they had been conducted after the parasite invaded. This was done so that records of absence collected prior to the parasite's introduction to a given area were not included in the comparison of prevalence between the native and introduced ranges. Dates of first introduction were used to determine inclusion of sites in the parasite’s introduced range in the meta-analysis. We assessed this primarily with the data in Kruse et al. (2011) and confirmed dates of earliest record by checking the literature for the year when parasitized crabs were first reported for a given area.

We used records of *L. panopaei* prevalence from 13 published studies, two unpublished data sets collected by the authors, one unpublished data set kindly shared by A.-L. Gehman, and this study. These ranged from the first record of *L. panopaei* invasion outside its native range in 1964 to data from the current study collected in 2015 (Van Engel, et al., 1966; Table S1.2). For *R. harrisii*, we found 132 records for 19 discrete sites in the native and 15 sites in the introduced ranges of *L. panopaei*. We note that these records are biased towards the Chesapeake Bay, with 86 records collected in the same 4 sites in Maryland over the course of an ongoing, unpublished study (GM Ruiz, pers. comm.). *L. panopaei* parasitism has been more widely-studied in *E. depressus*, and accordingly we identified substantially more sites for which prevalence estimates are available for this host. In all we found 95 records for 34 discrete sites in the native and 39 in the introduced ranges of *L. panopaei*.

**References (not included in main text):**

Alvarez, F., Hines, A. H., & Reaka-Kudla, M. L. (1995). The effects of parasitism by the barnacle *Loxothylacus panopaei* (Gissler) (Cirripedia: Rhizocephala) on growth and survival of the host crab *Rhithropanopeus harrisii* (Gould) (Brachyura: Xanthidae). *Journal of Experimental Marine Biology and Ecology*, 192, 221–232.

O’Shaughnessy, K. A., Freshwater, D. W., & Burge, E. J. (2014). Prevalence of the invasive rhizocephalan parasite *Loxothylacus panopaei* in *Eurypanopeus depressus* in South Carolina and genetic relationships of the parasite in North and South Carolina. *Journal of Parasitology*, 100, 447–454.

**Table S1.1**: Details on sampling sites. Italics indicate dates when collectors were initially deployed; no crab sampling was done at these times.

| **Code** | **Region** | **Estuary** |  |  |  |  |  |  |
| --- | --- | --- | --- | --- | --- | --- | --- | --- |
|  | *Site* | *Co-ordinates* | *Deployment* | | *Summer* | | *Fall* | |
|  |  |  | *Date* | *Sal* | *Date* | *Sal* | *Date* | *Sal* |
| **LA** | **Native** | **Terrebonne Bay, LA** |  |  |  |  |  |  |
|  | LA2 | 29.45662 , -90.47165 | *06/04/15* | 3.2 | 07/28/15 | 8.6 | 10/06/15 | 9.0 |
|  | LA3 | 29.41685 , -90.44833 | *06/04/15* | 8.0 | 07/29/15 | 9.8 | 10/06/15 | 11.6 |
|  | LA4 | 29.25413 , -90.66358 | *06/05/15* | 2.9 | 07/29/15 | 5.3 | 10/05/15 | 13.5 |
|  | LA5 | 29.42807 , -90.55515 |  |  | *07/29/15* | 4.1 | 10/05/15 | 5.1 |
| **AP** | **Native** | **Apalachicola River, FL** |  |  |  |  |  |  |
|  | AP1 | 29.76942 , -84.88147 | *06/06/15* | 6.3 | 07/31/15 | 11.1 | 10/08/15 | 18.3 |
|  | AP2 | 29.74003 , -84.89955 | *06/07/15* | 17.5 | 07/31/15 | 13.5 | 10/08/15 | 21.1 |
|  | AP3 | 29.69913 , -85.18900 | *06/08/15* | 17.5 | 08/01/15 | 31.5 |  |  |
|  | AP4 | 29.71357 , -85.00993 |  |  | *08/01/15* | 11.3 | 10/09/15 | 17.5 |
| **FP** | **Native** | **St. Lucie River, FL** |  |  |  |  |  |  |
|  | FP1 | 27.17457 , -80.25638 | *06/10/15* | 10.8 | 08/03/15 | 11.0 | 10/11/15 | 11.4 |
|  | FP2 | 27.19955 , -80.25837 | *06/10/15* | 16.0 | 08/03/15 | 14.5 |  |  |
|  | FP3 | 27.23268 , -80.30413 | *06/11/15* | 11.3 | 08/04/15 | 8.3 | 10/12/15 | 15.0 |
|  | FP4 | 27.27222 , -80.32172 |  |  | *08/04/15* | 1.0 | 10/11/15 | 5.8 |
| **ML** | **Introduced** | **Pellicer Creek, FL** |  |  |  |  |  |  |
|  | ML1 | 29.65382 , -81.24271 | *06/11/15* | 16.1 | 08/05/15 | 21.6 |  |  |
|  | ML2 | 29.66710 , -81.25735 | *07/10/15* | 10.5 | 08/06/15 | 14.2 | 10/13/15 | 13.2* |
|  | ML3 | 29.66816 , -81.26552 | *07/10/15* | 8.9 | 08/06/15 | 12.4 | 10/15/15 | 0.3* |
|  | ML5 | 29.65057 , -81.24140 |  |  | *08/06/15* | 26.6 | 10/14/15 | 19.0* |
|  | ML6 | 29.65059 , -81.24143 |  |  | *08/10/15* | nd | 10/15/15 | 1.0* |
| **SC** | **Introduced** | **Ashley River, SC** |  |  |  |  |  |  |
|  | SC1 | 32.84697 , -80.05077 | *06/14/15* | 2.1 | 08/08/15 | 7.5 | 10/16/15 | 0.2* |
|  | SC2 | 32.84523 , -80.03585 | *06/14/15* | 6.1 | 08/08/15 | 10.8 | 10/17/15 | 2.1* |
|  | SC3 | 32.83685 , -80.02238 | *06/14/15* | 13.1 | 08/07/15 | 20.8 |  |  |
|  | SC4 | 32.86605 , -80.06032 |  |  | *08/08/15* | 13.9 | 10/17/15 | 0.1* |
| **MD** | **Introduced** | **Chesapeake Bay, MD** |  |  |  |  |  |  |
|  | MD1 | 38.67720 , -76.17538 | *06/18/15* | 10.9 | 08/19/15 | 10.9 | 10/21/15 | 14.5 |
|  | MD2 | 38.41389 , -76.54883 | *06/29/15* | 9.9 | 8/22/15 | 11.9 | 11/03/15 | 14.3 |
|  | MD3 | 38.88613, -76.54142 | *06/11/15* | 9.8 | 8/21/15 | 9.4 | 11/06/15 | 16.5 |
| **NJ** | **Absent** | **Mullica River, NJ** |  |  |  |  |  |  |
|  | NJ1 | 39.59243 , -74.44165 | *06/22/15* | 7.4 | 08/12/15 | 14.5 | 11/20/15 | 8.0 |
|  | NJ2 | 39.54800 , -74.46155 | *06/22/15* | 16.1 | 08/12/15 | 18.8 |  |  |
|  | NJ3 | 39.53472 , -74.46325 | *06/23/15* | 9.7 | 08/12/15 | 13.1 | 11/20/15 | 9.6 |
| **MA** | **Absent** | **Moonakis & Mashpee Rivers, MA** | |  |  |  |  |  |
|  | MA1 | 41.60200 , -70.47193 | *06/30/15* | 17.9 | 09/10/15 | 25.3 |  |  |
|  | MA2 | 41.58335 , -70.51270 | *07/01/15* | 0.7 | 09/09/15 | 1.1 |  |  |
|  | MA3 | 41.58048 , -70.51240 | *07/01/15* | 13.2 | 09/10/15 | 11.3 |  |  |
| **NH** | **Absent** | **Squamscott River, NH** |  |  |  |  |  |  |
|  | NH1 | 43.05297 , -70.91240 | *07/03/15* | 20.3 | 09/12/15 | 27.9 |  |  |
|  | NH2 | 43.02855 , -70.93498 | *07/03/15* | 11.2 | 09/12/15 | 27.0 |  |  |
|  | NH3 | 43.04147 , -70.92712 | *07/03/15* | 18.3 | 09/12/15 | 21.3 |  |  |

* Sampling occurred just after a major rain / flooding event, and salinity is likely abnormally low.

nd = no data.

**Table S1.2**: Details on studies used in the meta-anaysis. References given in full in main text and Appendix S2. Status = status of the parasite in the region (native or introduced); Cutoff = minimum carapace width (in mm) of crabs reported in the study; N records = total number of records used for the species from the given study and estuary. NR = not reported: no apparent minimum cutoff, and no detail on minimum size observed given in the paper.

| **Study** | **Status** | **Cutoff** | **Estuary** | **Years** | **N records** |
| --- | --- | --- | --- | --- | --- |
| ***E. depressus*** | | | | | |
| Current study | Native | 5.8 | Apalachicola, FL  St. Lucie, FL | 2015 | 6  5 |
|  | Introduced | 5.8 | Chesapeake, MD | 2015 | 1 |
| Eash-Louks, et al., 2014 | Introduced | 6 | GTM, FL | 2005-2010 | 4 |
| Freeman, et al., 2013 | Introduced | 5 | Long Island, NY | 2012 | 1 |
| Gehman, et al., 2016 | Introduced | >4^a^ | St. Johns, FL  GTM, FL  Doboy, GA  Wassaw, GA  ACE Basin, SC  Winyah, SC  Lockwoods, NC  Masonboro, NC  Virginia Creek, NC  Back Sound, NC | 2010 | 1  1  1  1  1  1  1  1  1  1 |
| A-LM Gehman, unpublished | Native | NR | Alligator, FL  Cedar Keys, FL | 2010 | 1  1 |
| Hines, et al., 1997 | Native | 3^b^ | Apalachicola, FL  Alligator, FL  Steinhatchee, FL  Tampa, FL  Sarasota, FL  Gasparilla, FL  Naples, FL  Whipray, FL  Indian, FL | 1983-1993 | 1  1  1  1  1  1  1  1  8 |
|  | Introduced | 3^b^ | Winyah, SC  Back Sound, NC  Quinby, VA  Chincoteague, VA  Chesapeake, MD | 1983-1986 | 1  3  1  2  5 |
| Kroft, Blakeslee, 2016 | Introduced | >5^c^ | Long Island, NY | 2014 | 1 |
| Kruse, Hare, 2007 | Native | 5 | Cedar Keys, FL  Crystal, FL  Indian, FL | 2004-2005 | 2  1  4 |
|  | Introduced | 5 | Indian (north), FL  Halifax, FL  GTM, FL  St Johns, FL  St Marys, FL  Brunswick, GA  Doboy, GA | 2004-2005 | 1  1  2  2  1  1  1 |
| Kruse, et al., 2011 | Native | 5 | Terrebonne, LA  Mobile , AL  Mashes Sands, FL  Apalachicola, FL  Alligator, FL  Indian, FL | 2006 | 1  2  1  1  1  3 |
|  | Introduced | 5 | Indian, FL  Indian (north), FL | 2006 | 1  2 |
| O'Shaughnessy, Freshwater, Burge, 2014 | Introduced | 2.4^d^ | Winyah, SC  Little River, SC  Murrells, SC | 2012 | 13  13  13 |
| O'Shaughnessy, Harding, Burge 2014 | Introduced | 2.3^d^ | Winyah, SC | 2012 | 8 |
| The authors, unpublished | Introduced | 5.8 | Chesapeake, MD | 2004-2011 | 4 |
| Tolley, et al., 2006 | Native | NR | Caloosahatchee, FL  Estero , FL  Fakahatchee, FL | 2002 | 1  1  1 |
| Toscano, et al., 2014 | Introduced | NR | Winyah, SC | 2012 | 1 |
| Van Engel, et al., 1966 | Introduced | NR | Chesapeake, VA | 1964 | 1 |
| ***R. harrisii*** | | | | | |
| Current study | Native | 3.9 | Terrebonne, LA  Apalachicola, FL  St Lucie, FL | 2015 | 6  3  5 |
|  | Introduced | 3.9 | GTM, FL  Charleston, SC  Chesapeake, MD | 2015 | 5  4  6 |
| The authors, unpublished | Native | 1^a^ | Aransas, TX  Calcasieu, LA  Vermilion, LA  Terrebonne, LA  Apalachicola, FL | 2011 | 1  1  1  1  2 |
| Grosholz, Ruiz ,1995 | Introduced | NR | Chesapeake, MD | 1991-1992 | 4 |
| Hines, et al., 1997 | Native | 3^b^ | St Lucie, FL | 1983 | 1 |
|  | Introduced | 3^b^ | Pamlico, NC  Chesapeake, MD | 1993 | 1  1 |
| Kruse, Hare, 2007 | Native | 5 | Indian, FL | 2005 | 1 |
| Kruse, et al., 2011 | Native | 5 | Terrebonne, LA  Indian, FL | 2006 | 1  1 |
| The authors, unpublished | Introduced | 3.9 | Chesapeake, MD | 2004-2013 | 86 |
| Van Engel, et al., 1966 | Introduced | NR | Chesapeake, VA | 1964 | 1 |

^a^No minimum size cutoff was used: this is the minimum reported.

^b^No minimum size cutoff was used: very few crabs <5 mm CW and none <3 mm CW were reported.

^c^Larger crabs were preferentially collected; none was <5 mm CW.

^d^No minimum size cutoff was used: this is the minimum reported, and few crabs <4 mm CW were reported.

GTM = Guana Tolomato Matanzas estuary system.

**Appendix S2: Data Sources**

***Published meta-analysis data sources not listed in main text:***

O’Shaughnessy, K. A., Freshwater, D. W., & Burge, E. J. (2014). Prevalence of the invasive rhizocephalan parasite *Loxothylacus panopaei* in *Eurypanopeus depressus* in South Carolina and genetic relationships of the parasite in North and South Carolina. *Journal of Parasitology*, 100, 447–454.
